# Infarct quantification using 3D inversion recovery and 2D phase sensitive inversion recovery; validation in patients and ex vivo

**DOI:** 10.1186/1471-2261-13-110

**Published:** 2013-12-05

**Authors:** Robert Jablonowski, David Nordlund, Mikael Kanski, Joey Ubachs, Sasha Koul, Einar Heiberg, Henrik Engblom, David Erlinge, Håkan Arheden, Marcus Carlsson

**Affiliations:** 1 Department of Clinical Physiology, Lund University, Lund University Hospital, Lund, Sweden; 2 Department of Cardiology, Lund University, Lund University Hospital, Lund, Sweden; 3 Centre for Mathematical Sciences, Lund University, Lund, Sweden

## Abstract

**Background:**

Cardiovascular-MR (CMR) is the gold standard for quantifying myocardial infarction using late gadolinium enhancement (LGE) technique. Both 2D- and 3D-LGE-sequences are used in clinical practise and in clinical and experimental studies for infarct quantification. Therefore the aim of this study was to investigate if image acquisitions with 2D- and 3D-LGE show the same infarct size in patients and ex vivo.

**Methods:**

Twenty-six patients with previous myocardial infarction who underwent a CMR scan were included. Images were acquired 10-20 minutes after an injection of 0.2 mmol/kg gadolinium-based contrast agent. Two LGE-sequences, 3D-inversion recovery (IR) and 2D-phase-sensitive (PS) IR, were used in all patients to quantify infarction size. Furthermore, six pigs with reperfused infarction in the left anterior descending artery (40 minutes occlusion and 4 hours of reperfusion) were scanned with 2D- and 3D-LGE ex vivo. A high resolution T1-sequence was used as reference for the infarct quantification ex vivo. Spearman’s rank-order correlation, Wilcoxon matched pairs test and bias according to Bland-Altman was used for comparison of infarct size with different LGE-sequences.

**Results:**

There was no significant difference between the 2D- and 3D-LGE sequence in left ventricular mass (LVM) (2D: 115 ± 25 g; 3D: 117 ± 24 g: p = 0.35). Infarct size in vivo using 2D- and 3D-LGE showed high correlation and low bias for both LGE-sequences both in absolute volume of infarct (r = 0.97, bias 0.47 ± 2.1 ml) and infarct size as part of LVM (r = 0.94, bias 0.16 ± 2.0%). The 2D- and 3D-LGE-sequences ex vivo correlated well (r = 0.93, bias 0.67 ± 2.4%) for infarct size as part of the LVM. The IR LGE-sequences overestimated infarct size as part of the LVM ex vivo compared to the high resolution T1-sequence (bias 6.7 ± 3.0%, 7.3 ± 2.7% for 2D-PSIR and 3D-IR respectively, p < 0.05 for both).

**Conclusions:**

Infarct quantification with 2D- and 3D-LGE gives similar results in vivo with a very low bias. IR LGE-sequences optimized for in vivo use yield an overestimation of infarct size when used ex vivo.

## Background

Cardiac magnetic resonance (CMR) using late gadolinium enhancement (LGE) is the gold standard to assess myocardial viability in patients with ischemic heart disease. In LGE, infarcted myocardium yields a higher signal intensity compared to normal myocardium after the administration of an intravenous contrast agent [1-3]. Accurate quantitative assessment of the extent of scarring can guide treatment and is a strong predictive indicator for long term prognosis and restoration of contractile function following revascularisation therapy [4,5]. CMR can evaluate myocardial infarction in both the acute and chronic phase and it is superior to other imaging modalities in its higher spatial resolution and high contrast between infarcted tissue and viable myocardium [6,7]. The reference standard for clinical LGE-CMR imaging has been the breath-hold two-dimensional inversion recovery (2D-IR) gradient echo (GRE) sequence with magnitude reconstruction [8]. Images are acquired slice by slice with the need to manually adjust the inversion time (TI) to optimally null the myocardium and require relative long acquisition times [8]. The problem of finding the right TI can be solved by utilizing a phase-sensitive-inversion recovery (PSIR) sequence without the need to precisely choose the right TI to null the myocardium but instead using a fixed TI across the scanning range [9]. Infarct quantification using a 2D-IR sequence shows good agreement compared to a 2D-PSIR sequence [9]. Because the 2D-LGE sequences requires a relative long acquisition time a three-dimensional inversion recovery (3D-IR) GRE sequence has been developed to shorten the scanning time with multiple slices being acquired in a single breathold [10]. Both 2D- and 3D-LGE-sequences are used in clinical practice and in clinical and experimental studies for infarct quantification both in vivo and ex vivo. Previous studies compared infarct quantification with different 2D-IR and 3D-IR sequences and showed good agreement both in patients [11,12] and in vivo in animals [10,12]. However, 3D-IR has not been validated against 2D-PSIR in patients nor compared to a high resolution T1 weighted (T1w) reference sequence ex vivo. Therefore the aim of this study was 1) to investigate the agreement between 2D-PSIR and a 3D-IR for measurement of infarct size in patients and 2) to compare 2D-PSIR and 3D-IR with a reference high resolution T1w sequence in an experimental infarct model.

## Methods

### Ethics

The study conforms to the Guide for the Care and Use of Laboratory animals US National Institute of Health (NIH Publication No. 85-23, revised 1996) and was in compliance with the Helsinki Declaration. The study was approved by the Ethics Committee of Lund University, Lund, Sweden and patients have given informed consent.

### Study population

Twenty-six patients (20 males and 6 females, age 62 ± 9 years and 57 ± 14 years) with a documented ST-elevation myocardial infarction (STEMI) treated with percutaneous coronary intervention (PCI) referred for viability assessment were prospectively included. A total of 19 patients had an acute myocardial infarction (CMR < 30 days after infarction) and 7 patients had a non-acute infarction (CMR > 30 days after infarction) [12]. All patients underwent a CMR scan including two different LGE-sequences.

### Ex vivo

For ex vivo imaging, a previously described pig model was used [13,14]. After overnight fasting with free access to water the animals were premedicated with ketamine 15 mg/kg (Ketaminol, Intervet, Danderyd, Sweden) and xylazin 2 mg/kg intramuscularly (Rompun, Bayer AG, Leverkusen, Germany). Anesthesia was induced with thiopental 12.5 mg/kg (Pentothal, Abbot, Stockholm, Sweden) and infusion of fentanyl (Fentanyl, Pharmalink AB, Stockholm, Sweden). Six pigs weighing 30-40 kg underwent 40 minutes of occlusion of the left anterior descending artery (LAD) using a balloon-tipped catheter, followed by 4 hours of reperfusion. Thirty minutes before explantation of the heart, a single dose of MRI contrast agent was intravenously administrated (0.2 mmol/kg Dotarem, Guerbet, Roissy, France). Following explantation the hearts were suspended in a plastic container with the atria excised and filled with balloons containing deuterated water in the right and left ventricle for ex vivo imaging.

### MR-imaging

#### Patients

Imaging was performed at 1.5 T (Philips Achieva, Best, Netherlands) using a 32 channel coil. Cine imaging was performed in all patients. ECG-triggered LGE imaging was performed with both a 2D-PSIR and a 3D-IR GRE sequence acquired during mid diastole during end-expiratory breath-hold. Both magnitude and phase-sensitive images were acquired with the 2D-LGE sequence. Short-axis slices covering the entire left ventricle from base to apex and three different long-axis projections were collected. The 2D-LGE sequence was acquired slice by slice during breathold whereas the 3D-LGE sequence was acquired 5 slices per breathold, 10-20 minutes after intravenous administration of 0.2 mmol/kg gadolinium based MR contrast agent (Dotarem, Guerbet, Roissy, France). 2D-PSIR was acquired first in 10 patients, and 3D-IR acquisition was performed first in 16 patients. No additional contrast was administered between imaging with the different LGE-sequences. Typical image parameters for the 3D-IR sequence were: echo time 1.3 ms, effective repetition time every heartbeat, flip angle 15°, slice gap 0 mm, slice thickness 8 mm and inplane resolution 1.5 × 1.5. For the 2D-PSIR sequence the typical image parameters were: echo time 4 ms, effective repetition time every second heartbeat, flip angle 25°, slice gap 0 mm, slice thickness 8 mm and in-plane resolution 1.4 × 1.4. The inversion time was chosen to optimally null the myocardium and was typically 280 ms for 3D-IR and between 300-350 ms for 2D-PSIR.

#### Ex vivo

Ex vivo imaging with 2D-PSIR, 3D-IR and a high resolution T1w sequence was performed using a head coil covering the left ventricle from the base to the apex with the heart suspended in a plastic container. Acquisition with the T1w sequence typically resulted in 200-220 image slices per heart with a acquisition time of 30-40 minutes. Typical sequence parameters for the T1w sequence were: echo time 3.2 ms, repetition time 20 ms, flip angle 70°, slice gap 0 mm, slice thickness 0.5 mm, inplane resolution 0.5 × 0.5 mm. The sequence parameters for the 2D-PSIR and 3D IR sequences were the same as in patients using a simulated ECG with heart rate 60/min. The spatial resolution for 2D-PSIR was 0.6 × 0.6 × 4 mm and for 3D-IR 1 × 1 × 4 mm.

### Image analysis

All MR images were evaluated by two blinded observers using the software Segment (Segment v1.9 R2823, http://segment.heiberg.se) [15]. The left ventricular mass (LVM), in patients and ex vivo, was quantified on LGE-images and on high resolution T1w images by delineating the endo- and epicardium. The delineation of the endocardium included papillary muscles and trabeculations as left ventricular volume. The infarcts on 2D- and 3D-LGE images in patients and ex vivo were quantified using a semi-automatic weighted algorithm with manual corrections as previously described [16]. Infarct transmurality was calculated for both sequences by assessing the radial extent of the infarction between the endocardial and epicardial borders of the myocardial wall around the circumference at 4.5° intervals of the short-axis LGE images [17]. The mean transmurality of the infarct in all slices with hyperenhancement was calculated. In the high resolution T1w sequence scarred myocardium was quantified by manually drawing regions of interest (ROI) in both scarred and normal myocardium and using a threshold in signal intensity of 8 standard deviations (SD) to differentiate between normal and scarred myocardium [18]. Manual corrections were also applied when necessary for example in the setting of microvascular obstruction.

### Statistical analysis

Calculations and statistics were performed using Graph Pad Prism 5.0 software (Graph Pad Software, Inc., La Jolla, CA, USA). Results are expressed as mean ± SD and Spearman’s rank-order correlation, Wilcoxon matched pairs test and bias according to Bland-Altman was used for comparison of infarct size with different LGE-sequences in patients and ex vivo. Statistical significance was defined as results with a p < 0.05.

## Results

### Patient studies

Three patients were excluded because of poor image quality due to breathing artefacts and inadequate nulling of the myocardium. A total of 23 patients were included in the study and all patients showed myocardial hyperenhancement on both 2D- and 3D-LGE sequences.

**Figure 1:**
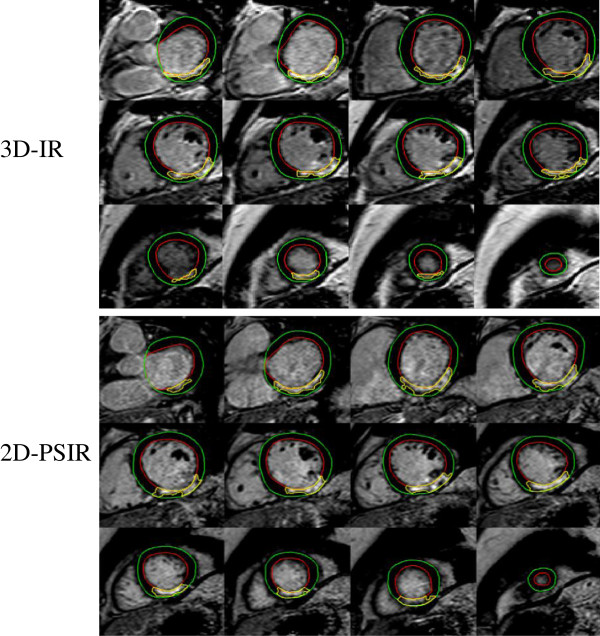
**Patient LGE-imaging.** Representative short axis LGE-images in one patient with myocardial scarring from base to apex (advancing from left to right). Top panels show 3D-IR images and bottom panels show 2D-PSIR images. (The green line denotes the epicardial border and the red line denotes the endocardial border. The infarct is delineated in yellow.)

**Figure 2:**
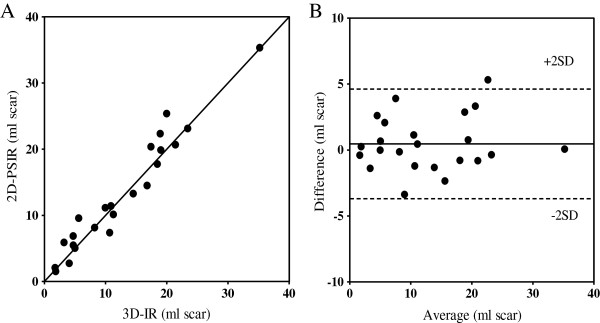
**Patient infarct quantification.** Agreement between 2D-PSIR and 3D-IR in absolute infarct volume in patients. **(A)** 2D-PSIR versus 3D-IR (r = 0.97) and the line of identity **(B)** The limits of agreement between the two LGE-techniques. The difference between the two methods was 0.47 ± 2.1 ml scar. Solid line = mean difference; dashed lines = ± 2 SD.

Figure 1 illustrates short axis MR images acquired in a representative patient with myocardial scarring with 2D-LGE, both magnitude and phase-sensitive images, and 3D-IR. There was no significant difference in mean LVM between the 2D- and 3D-LGE sequence (2D-PSIR: 115 ± 25 g: 3D-IR: 117 ± 24 g: p = 0.35). All patients were in sinus rhythm during imaging and the heart rates were similar for both acquisitions (2D-PSIR: 62 ± 2 beats/min; 3D-IR 63 ± 2 beats/min, r = 0.97, bias = -0.08 ± 2.2, p = 0.85). Infarct size in vivo using 2D-PSIR and 3D-IR showed high correlation and low bias for both LGE-sequences (Figure 2) both in absolute volume of infarct (r = 0.97, bias 0.47 ± 2.1 ml) and infarct size as part of the LVM (r = 0.94, bias 0.16 ± 2.0%). Interobserver variability for infarct volume was -0.78 ± 2.8 ml for 2D-PSIR and 0.95 ± 2.9 ml for 3D-IR. Mean infarct transmurality did not differ significantly between the two sequences (2D-PSIR: 44.1 ± 2.6% and 3D-IR: 44.6 ± 2.4%, r =0.78, bias 0.52 ± 8.4, p = 0.77). Furthermore, there was good agreement of left ventricular volumes quantified on 2D-PSIR and 3D-IR (175 ± 15 ml versus 170 ± 15 ml respectively, r = 0.88, bias = 5.5 ± 21 ml, p = 0.23). The acquisition time was on average 225 seconds on 2D-PSIR and 45 seconds on 3D-IR, p < 0.05.

### Animal studies

**Figure 3:**
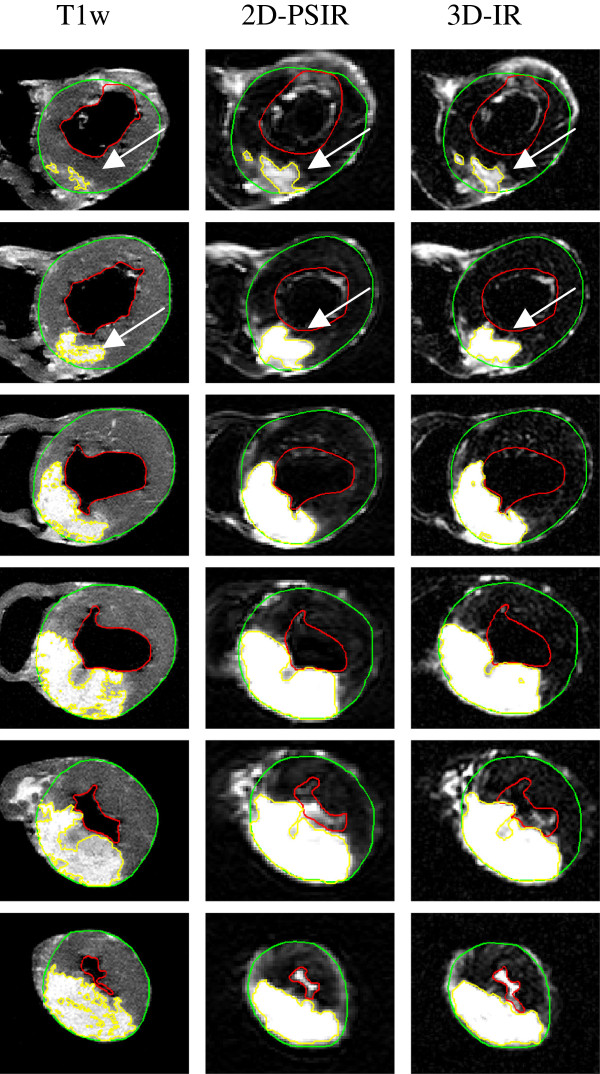
**Ex vivo imaging.** Ex vivo short axis images in a representative pig heart from base (top) to apex (bottom). Left column shows high resolution T1 weighted images, middle column shows 2D-PSIR images and right column show 3D-IR images. The green line denotes the epicardial border and the red line denotes the endocardial border. The infarct is delineated in yellow. Note the difference in infarct size between the T1w-sequence and the IR-LGE sequences as showed by the white arrows in the top two rows.

**Figure 4:**
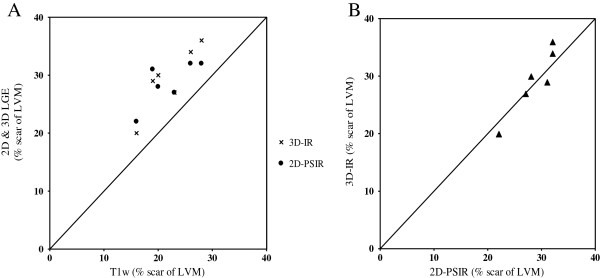
**Ex vivo infarct quantification. (A)** Agreement between the high resolution T1 weighted sequence compared to 2D-PSIR and 3D-IR ex vivo. The bias was 6.7 ± 3.0% for 2D-PSIR sequence and 7.3 ± 2.7% for the 3D-IR sequence (p < 0.05 for both). **(B)** Agreement between the 2D-PSIR and 3D-IR sequence ex vivo. Each black triangle represents one pig. The bias was 0.67 ± 2.4% and r = 0.93. Solid line = line of identity in both panel **A** and **B**.

Figure 3 illustrates representative short axis images ex vivo with corresponding epicardial, endocardial and scar delineations on 2D- and 3D-LGE and a high resolution T1w sequence. The 2D-PSIR and 3D-IR sequences ex vivo correlated well (r = 0.93, bias 0.67 ± 2.4%) for infarct size as part of the LVM (Figure 4). The IR LGE-sequences overestimated infarct size as part of the LVM when used ex vivo compared to the high resolution T1-sequence (bias 6.7 ± 3.0%, 7.3 ± 2.7% for 2D-PSIR and 3D-IR respectively, p < 0.05 for both, Figure 4).

## Discussion

This study has demonstrated that myocardial infarct quantification with a 2D-PSIR and a 3D-IR sequence show good agreement in patients with both acute and non-acute infarcts. This allows for the sequences to be used interchangeably in a clinical setting as well as for clinical studies using LGE-MR. The current study also demonstrates that in an experimental setting both the 2D- and the 3D-LGE-sequence overestimate infarct size compared to a high resolution T1 weighted sequence ex vivo.

### Clinical 2D- vs 3D-LGE

The use of the clinical standard for viability imaging, 2D-IR-GRE, requires 10–16 slices each acquired over 14 heartbeats to assess the entire left ventricle. The 2D image is acquired on every second heartbeat which enables the M_0_ signal to recover before a new IR-pulse is given and is therefore less sensitive to arrhythmias [1,8]. Imaging with 3D-LGE yield higher or similar signal-to-noise-ratio (SNR) and contrast-to-noise-ratio (CNR) compared to 2D-LGE [19,20]. Since the 3D-sequence triggers on every heartbeat it renders a shorter acquisition time and it requires fewer breatholds for the patient but varying R-R intervals and arrhythmias cause decreased image quality. Previous studies comparing infarct size in patients have shown good agreement between different 2D- and 3D-LGE sequences, both using a semiautomatic algorithm for LGE-quantification [12] and visual assessment [21-23]. Dewey *et al* compared a 2D-IR and a single breathold 3D IR sequence in 13 patients and found good agreement between quantified infarct volumes (bias 0.3 cm^3^ ± 3.1) similar to what we found in the current study [10]. Peukert *et al*[11] compared infarct quantification on 3D IR FLASH and 2D-IR FLASH in patients demonstrating good agreement and correlation (-0.1 ± 2.1 ml infarct, r = 0.98). Furthermore, Goetti *et al*[12] compared 2D- and 3D-IR GRE in patients with both acute, sub-acute and chronic infarction and found good agreement in all three groups with a mean difference of 0.26 g infarct ± 2.88. In the current study 2D-PSIR GRE was compared to 3D-IR GRE in patients with acute and chronic myocardial infarction demonstrating good agreement and correlation (0.47 ± 2.1 ml infarct, r = 0.97) in line with previous studies comparing 3D-LGE to conventional 2D-IR GRE.

We used left ventricular mass on LGE-CMR to express infarct size as percentage of LVM and LVM on 2D-PSIR and 3D-IR agreed well. Stephensen *et al* showed [24] that quantification of LVM on steady state free precession (SSFP) images will yield a slightly higher LVM (bias: 5.0 ± 6.7 g) compared with quantification on LGE-CMR.

### Ex vivo infarct quantification

Previous studies have shown good agreement between in vivo quantification of myocardial infarction on 2D and 3D-LGE in pigs [10,11]. Furthermore, the studies showed good agreement with histochemical TTC in infarct transmurality and volume quantification using visual assessment [10,11]. In the current study, we compared 2D- PSIR and 3D-IR imaging ex vivo with a high resolution T1w sequence. The high resolution T1w sequence used in our study has been validated to histopathology staining with TTC [2] and has the advantage of imaging enhancement in the same position and with the same image analysis software. In our study, we found an overestimation of infarct volume with both 2D- and 3D LGE compared to the reference standard, the high resolution T1w sequence. This difference from previous studies can possibly be attributed to that we used an objective semi-automatic method for infarct quantification. The method for automatic quantification of ex vivo high resolution imaging using a threshold of 8 SD has been validated by Heiberg *et al*[18]. The authors demonstrated that 7-9 SD provided similar results but lower SD:s overestimated infarct size compared to manual delineations by experts. Based on the result from the current study clinical IR-LGE sequences optimized for in vivo use should not be used in the ex vivo setting.

### Implications for multicenter studies

In clinical multicenter trials where infarct quantification is performed following revascularization or other therapies, the assessment of myocardial salvage is a predictor of therapy outcome [25,26]. Myocardial salvage is defined as how much of the myocardium at risk is saved following revascularization therapies compared to final infarct size [27]. CMR can depict the myocardium at risk, using either T2-imaging or contrast-enhanced SSFP (CE-SSFP), with the same accuracy as single photon emission computed tomography [25,26]. This enables CMR to assess myocardial salvage during one examination. In multicenter trials the equipment and used techniques should be as similar as possible. However, having the same MR scanner as a prerequisite would exclude many potential centers. The implication of this study is that infarct size quantification on LGE-MRI using two different sequences, 2D PSIR and 3D IR, can be pooled together. This study does not address the question of using different scanners for LGE imaging. However the study supports that 2D-PSIR GRE or 3D-IR GRE can be used interchangeably on the current scanner used (Philips Achieva 1.5 T) and possibly on other scanners in order to enroll larger patient cohorts.

### Limitations

The order of the acquisitions was not randomized but in a subset of patients 2D-LGE imaging was performed before 3D-LGE and vice versa. However, the images are comparable regarding hyperenhancement since it has been shown that normal myocardium is in equilibrium with the blood pool only minutes after contrast bolus administration and remains constant up to 25 minutes [28]. In the experimental setting we did not perform histopathological staining to compare with the IR-LGE and T1w sequences but the high resolution T1w sequence has been validated against TTC for infarct size quantification [2].

## Conclusions

In summary, infarct quantification with 2D PSIR- and 3D-IR provides similar results in vivo with a low bias and the two sequences can be used interchangeably. IR LGE-sequences optimized for in vivo use yield an overestimation of infarct size ex vivo.

## Abbreviations

CMR: Cardiac magnetic resonance imaging; LGE: Late gadolinium enhancement; IR: Inversion recovery; PSIR: Phase sensitive inversion recovery; GRE: Gradient echo; TI: inversion time; T1w: T1 weighted; SNR: Signal-to-noise-ratio; CNR: Contrast-to-noise-ratio; LAD: Left anterior descending artery; TTC: Triphenyltetrazolium chloride; CE-SSFP: Contrast-enhanced steady state free precession; ROI: Region of interest; SD: Standard deviation.

## Competing interests

The authors declare that they have no competing interests.

## Authors’ contributions

The authors have contributed as follows: RJ participated in the animal experiments, performed the analysis and drafted the manuscript. DN analyzed clinical data. SK, MK and JU participated in the animal experiments. HA, DE, EH, HE and MC conceived and designed the study. All authors read, critically revised and approved the final manuscript.

## Pre-publication history

The pre-publication history for this paper can be accessed here:

http://www.biomedcentral.com/1471-2261/13/110/prepub

## References

[B1] Simonetti OP Kim RJ Fieno DS Hillenbrand HB Wu E Bundy JM Finn JP Judd RM An improved MR imaging technique for the visualization of myocardial infarction Radiology 2001 13 215 223 10.1148/radiology.218.1.r01ja50215 11152805

[B2] Kim RJ Fieno DS Parrish TB Harris K Chen EL Simonetti O Bundy J Finn JP Klocke FJ Judd RM Relationship of MRI delayed contrast enhancement to irreversible injury Circulation 1999 13 1992 2002 10.1161/01.CIR.100.19.1992 10556226

[B3] Carlsson M Arheden H Higgins CB Saeed M Magnetic resonance imaging as a potential gold standard for infarct quantification J Electrocardiol 2008 13 614 620 10.1016/j.jelectrocard.2008.06.010 18817927

[B4] Selvanayagam JB Kardos A Francis JM Wiesmann F Petersen SE Taggart DP Neubauer S Value of delayed-enhancement cardiovascularmagnetic resonance imaging inpredicting myocardial viability after surgical revascularization Circulation 2004 13 1535 1541 10.1161/01.CIR.0000142045.22628.74 15353496

[B5] Kim RJ Wu E Rafael A Chen EL Parker MA Simonetti O Klocke FJ Bonow RO Judd RM The use of contrast-enhanced magnetic resonance imaging to identify reversible myocardial dysfunction N Engl J Med 2000 13 1445 1453 10.1056/NEJM200011163432003 11078769

[B6] Wagner A Mahrholdt H Holly TA Elliott MD Regenfus M Parker M Klocke FJ Bonow RO Kim RJ Judd RM Contrast-enhanced MRI and routine single photon emission computed tomography (SPECT) perfusion imaging for detection of subendocardial myocardial infarcts: an imaging study Lancet 2003 13 374 379 10.1016/S0140-6736(03)12389-6 12573373

[B7] Lund GK Stork A Saeed M Bansmann MP Gerken JH Müller V Mester J Higgins CB Adam G Meinertz T Acute myocardial infarction: evaluation with first-pass enhancement and delayed enhancement MR imaging compared with 201Tl SPECT imaging Radiology 2004 13 49 57 10.1148/radiol.2321031127 15166320

[B8] Kim RJ Shah DJ Judd RM How we perform delayed enhancement imaging J Cardiovasc Magn Reson 2003 13 505 514 10.1081/JCMR-120022267 12882082

[B9] Kellman P Arai AE McVeigh ER Aletras AH Phase-sensitive inversion recovery for detecting myocardial infarction using gadolinium-delayed hyperenhancement Magn Reson Med 2002 13 372 383 10.1002/mrm.10051 11810682 PMC2041905

[B10] Dewey M Laule M Taupitz M Kaufels N Hamm B Kivelitz D Myocardial viability: assessment with three-dimensional MR imaging in pigs and patients Radiology 2006 13 703 709 10.1148/radiol.2393050586 16641341

[B11] Peukert D Laule M Taupitz M Kaufels N Hamm B Dewey M 3D and 2D delayed-enhancement magnetic resonance imaging for detection of myocardial infarction: preclinical and clinical results Acad Radiol 2007 13 788 794 10.1016/j.acra.2007.03.006 17574129

[B12] Goetti R Kozerke S Donati OF Surder D Stolzmann P Kaufmann PA Luscher TF Corti R Manka R Acute, subacute and chronic myocardial infarction: quantitative comparison of 2D and 3D late gadolinium enhancement MR imaging Radiology 2011 13 704 711 10.1148/radiol.11102216 21467254

[B13] van der Pals J Koul S Andersson P Götberg M Ubachs JFA Kanski M Arheden H Olivecrona GK Larsson B Erlinge D Treatment with the C5a receptor antagonist ADC-1004 reduces myocardial infarction in a porcine ischemia-reperfusion model BMC Cardiovasc Disord 2010 13 45 10.1186/1471-2261-10-45 20875134 PMC2955599

[B14] Götberg M Olivecrona GK Engblom H Ugander M van der Pals J Heiberg E Arheden H Erlinge D Rapid short-duration hypothermia with cold saline and endovascular cooling before reperfusion reduces microvascular obstruction and myocardial infarct size BMC Cardiovasc Disord 2008 13 7 10.1186/1471-2261-8-7 18402663 PMC2323360

[B15] Heiberg E Sjogren J Ugander M Carlsson M Engblom H Arheden H Design and validation of Segment-freely available software for cardiovascular image analysis BMC Med Imaging 2010 13 1 10.1186/1471-2342-10-1 20064248 PMC2822815

[B16] Heiberg E Engblom H Engvall J Hedström E Ugander M Arheden H Semi-authomatic quantification of myocardial infarction from delayed contrast enhanced magnetic resonance imaging Scand Cardiovasc J 2005 13 267 275 10.1080/14017430500340543 16269396

[B17] Engblom H Hedstrom E Heiberg E Wagner GS Pahlm O Arheden H Size and transmural extent of first-time reperfused myocardial infarction assessed by cardiac magnetic resonance can be estimated by 12-lead electrocardiogram Am Heart J 2005 13 920 16290962 10.1016/j.ahj.2005.07.022

[B18] Heiberg E Ugander M Engblom H Gotberg M Olivecrona GK Erlinge D Arheden H Automated quantification of myocardial infarction from MR images by accounting for partial volume effects: animal, phantom, and human study Radiology 2008 13 581 588 10.1148/radiol.2461062164 18055873

[B19] Tatli S Zou KH Fruitman M Reynolds GH Foo T Kwong R Yucel KE Three-dimensional magnetic resonance imaging technique for myocardial-delayed hyperenhancement: a comparison with the two-dimensional technique J Cardiovasc Magn Reson 2004 13 378 382 10.1002/jmri.20124 15332243

[B20] Foo TK Stanley DW Castillo E Rochitte CE Wang Y Lima JA Bluemke DA Wu KC Myocardial viability: breath-hold 3D MR imaging of delayed hyperenhancement with variable sampling in time Radiology 2004 13 845 851 10.1148/radiol.2303021411 14990846

[B21] Kino A Zuehlsdorff S Sheehan JJ Weale PJ Carroll TJ Jerecic R Carr JC Three-dimensional phase-sensitive inversion-recovery turbo FLASH sequence for the evaluation of left ventricular myocardial scar Am J Roentgenol 2009 13 381 388 10.2214/AJR.08.1952 19843715

[B22] Nguyen TD Spincemaille P Weinsaft JW Ho BY Cham MD Prince MR Wang Y A fast navigator-gated 3D sequence for delayed Enhancement MRI of the myocardium: comparison with breathhold 2D Imaging J Magn Reson Imaging 2008 13 802 808 10.1002/jmri.21296 18302233

[B23] Kühl HP Papavasiliu TS Beek AM Hofman MB Heusen NS van Rossum AC Myocardial viability: rapid assessment with delayed contrast enhanced MR imaging with three-dimensional inversion-recovery prepared pulse sequence Radiology 2004 13 576 582 10.1148/radiol.2302021120 14688401

[B24] Stephensen SS Carlsson M Ugander M Engblom H Olivecrona G Erlinge D Arheden H Agreement of left ventricular mass in steady state free precession and delayed enhancement MR images: implications for quantification of fibrosis in congenital and ischemic heart disease BMC Med Imaging 2010 13 4 10.1186/1471-2342-10-4 20096134 PMC2881013

[B25] Carlsson M Ubachs JF Hedstrom E Heiberg E Jovinge S Arheden H Myocardium at risk after acute infarction in humans on cardiac magnetic resonance: quantitative assessment during follow-up and validation with singe-photon emission computed tomography JACC Cardivasc Imaging 2009 13 569 576 10.1016/j.jcmg.2008.11.018 19442942

[B26] Sörensson P Heiberg E Saleh N Bouvier F Caidahl K Tornvall P Rydén L Pernow J Arheden H Assessment of myocardium at risk with contrast enhanced steady-state free precession cine cardiovascular magnetic resonance compared to singe-photon emission computed tomography J Cardiovasc Magn Reson 2010 13 25 20433716 10.1186/1532-429X-12-25 PMC2885384

[B27] Hedstrom E Engblom H Frogner F Astrom-Olsson K Ohlin H Jovinge S Arheden H Infarct evolution in man studied in patients with first-time coronary occlusion in comparison to different species - implications for assessment of myocardial salvage J Cardiovasc Magn Reson 2009 13 38 10.1186/1532-429X-11-38 19775428 PMC2758877

[B28] Ugander M Oki AJ Hsu L-Y Kellman P Greiser A Aletras AH Sibley C Chen MY Bandettini PW Arai AE Extracellular volume imaging by MRI provides insight into overt and subclinical myocardial pathology Eur Heart J 2012 13 1268 1278 10.1093/eurheartj/ehr481 22279111 PMC3350985

